# Immune outcomes of Zika virus infection in nonhuman primates

**DOI:** 10.1038/s41598-020-69978-w

**Published:** 2020-08-03

**Authors:** Blake Schouest, Marissa Fahlberg, Elizabeth A. Scheef, Matthew J. Ward, Kyra Headrick, Dawn M. Szeltner, Robert V. Blair, Margaret H. Gilbert, Lara A. Doyle-Meyers, Victoria W. Danner, Myrna C. Bonaldo, Dawn M. Wesson, Antonito T. Panganiban, Nicholas J. Maness

**Affiliations:** 1 Biomedical Sciences Training Program, Tulane University School of Medicine, New Orleans, LA USA; 2 Division of Microbiology, Tulane National Primate Research Center, Covington, LA USA; 3 Division of Immunology, Tulane National Primate Research Center, Covington, LA USA; 4 School of Public Health and Tropical Medicine, Tulane University, New Orleans, LA USA; 5 Division of Comparative Pathology, Tulane National Primate Research Center, Covington, LA USA; 6 Division of Veterinary Medicine, Tulane National Primate Research Center, Covington, LA USA; 7 Laboratório de Biologia Molecular de Flavivírus, Instituto Oswaldo Cruz, Fiocruz, Rio de Janeiro, RJ Brazil; 8 Department of Microbiology and Immunology, Tulane University School of Medicine, New Orleans, LA USA

**Keywords:** Infectious diseases, Viral infection, Viral host response

## Abstract

Although the Zika virus (ZIKV) epidemic is subsiding, immune responses that are important for controlling acute infection have not been definitively characterized. Nonhuman primate (NHP) models were rapidly developed to understand the disease and to test vaccines, and these models have since provided an understanding of the immune responses that correlate with protection during natural infection and vaccination. Here, we infected a small group of male rhesus (*Macaca mulatta*) and cynomolgus (*Macaca fascicularis*) macaques with a minimally passaged Brazilian ZIKV isolate and used multicolor flow cytometry and transcriptional profiling to describe early immune patterns following infection. We found evidence of strong innate antiviral responses together with induction of neutralizing antibodies and T cell responses. We also assessed the relative importance of CD8 T cells in controlling infection by carrying out CD8 T cell depletion in an additional two animals of each species. CD8 depletion appeared to dysregulate early antiviral responses and possibly increase viral persistence, but the absence of CD8 T cells ultimately did not impair control of the virus. Together, these data describe immunological trends in two NHP species during acute ZIKV infection, providing an account of early responses that may be important in controlling infection.

## Introduction

Zika virus (ZIKV) has been a known pathogen for over half a century^1^^,^ but severe disease manifestations were not directly associated with the virus for most of its history. The recent outbreaks of ZIKV in the Western hemisphere became linked to neurologic and congenital syndromes that have provided strong incentives to develop prophylactic and therapeutic countermeasures. Several vaccine candidates spanning multiple platforms have performed well in preclinical studies and have advanced to clinical trials, but the waning of the epidemic has precluded efficacy testing in large populations^2,3^. Additionally, an understanding of the immune responses that provide protection during natural infection is not yet complete, which also complicates our ability to evaluate whether vaccines will replicate these protective responses.

The immunology of ZIKV infection has been most extensively studied in mice, and murine studies have shown the critical importance of type-I interferon (IFN) in limiting disease together with the protective roles of humoral and T cell responses^4–7^. A growing body of literature also describes immune responses following ZIKV infection in nonhuman primates (NHPs), which are often used in preclinical vaccine studies^8^ given their genetic and immunologic similarities to humans. Multiple species of macaques have been utilized, and these models replicate clinical aspects of viral infection and disease^9–13^. However, the relative importance of innate and adaptive responses in controlling infection in monkeys is only beginning to be explored in depth, and this data is an important open question for the evaluation of candidate vaccines.

Here, we carried out a descriptive analysis of early immune responses following ZIKV infection in the recently developed rhesus^9–13^ and cynomolgus^13,14^ macaque models. We tracked viremia and viral dissemination and assessed the activation of innate and adaptive immune responses using flow cytometry and transcriptomic profiling. We also evaluated the relative importance of CD8 T cells in controlling infection by depleting these cells in a limited number of animals of each species.

## Methods

### Animal experiments

The adult male Indian origin rhesus macaques (*Macaca mulatta*; mean age: 9.6 years; range: 6.7–11.9 years) and cynomolgus macaques (*Macaca fascicularis*; mean age: 8.8 years; range: 8.7–9.2 years) utilized in this study were housed at the Tulane National Primate Research Center (TNPRC). The TNPRC is fully accredited by AAALAC International (Association for the Assessment and Accreditation of Laboratory Animal Care), Animal Welfare Assurance No. A3180-01. Animals were cared for in accordance with the NRC Guide for the Care and Use of Laboratory Animals and the Animal Welfare Act. Animal experiments were approved by the Institutional Animal Care and Use Committee of Tulane University (protocol P0367).

Two rhesus and two cynomolgus macaques were subcutaneously infected with 10^4^ plaque forming units (PFU) of a minimally passaged Brazilian ZIKV isolate^15^ at 0 days post-infection (dpi). The cynomolgus macaque C46456 was splenectomized 9 months and 19 days prior to inoculation with ZIKV as part of a previous study. Whole blood, cerebrospinal fluid (CSF), and semen were obtained from animals throughout infection. Peripheral blood mononuclear cells (PBMCs) were isolated from the blood of rhesus macaques using SepMate tubes (Stemcell Technologies) according to the manufacturer’s protocol or from the blood of cynomolgus macaques using Lymphoprep (Stemcell Technologies) for standard density gradient centrifugation. At necropsy, the indicated tissues were collected and snap-frozen. Rhesus macaques were euthanized at 30 dpi, and cynomolgus macaques were euthanized at 15 dpi.

An additional two rhesus macaques (R25671 and R64357) and two cynomolgus macaques (C78777 and C18942) were depleted of CD8+ lymphocytes by administration of the anti-CD8α antibody MT807R1 (NHP Reagent Resource; https://www.nhpreagents.org)^16^. The initial subcutaneous administration of 10 mg/kg at 14 days pre-infection was followed by three intravenous administrations of 5 mg/kg at 11, 7, and 5 days pre-infection. C84545 was treated with the irrelevant control antibody anti-desmipramine (NHP Reagent Resource, https://www.nhpreagents.org) at the same dosages and time intervals pre-infection. Following antibody administration, animals were infected with the same strain and dose of ZIKV described above.

### Virus quantification

Viral RNA was extracted from serum and CSF using the High Pure Viral RNA Kit (Roche). Semen, as well as the indicated lymphoid, reproductive, gastrointestinal (GI), and neural tissues were homogenized in Qiazol (Qiagen) using either disposable tissue grinders (Fisherbrand) or a TissueRuptor (Qiagen), and RNA was isolated using the RNeasy Lipid Tissue Mini Kit (Qiagen). Viral RNA from body fluids and tissues was quantified using qRT-PCR as described previously^12^.

### Antiviral gene expression assays

2.5 ml whole blood was drawn from each animal at 0, 1, 3, and 15 dpi into PAXgene blood RNA tubes (PreAnalytiX) and equilibrated to − 80 °C as per the manufacturer’s protocol. RNA was extracted from blood samples using the PAXgene blood RNA kit (PreAnalytiX), and cDNA was synthesized using the RT2 First Strand Kit (Qiagen). Transcriptional profiles of immune signaling were generated using the nCounter NHP Immunology Panel of 770 macaque immune response genes (NanoString Technologies). In whole blood, transcriptional responses were assessed at 3 dpi relative to expression levels pre-infection using nSolver software v4.0 (NanoString Technologies). Differential gene expression was carried out using limma^17^^,^ and heatmaps of fold change data were generated using the pheatmap package (version 1.0.12) in R (version 3.6.1).

To identify the contribution of monocytes to antiviral signaling in blood, the CD14 MicroBead kit (Miltenyi Biotec) was used to sort CD14+ monocytes from the PBMCs of cynomolgus macaques at multiple timepoints. Due to limited sample availability, similar experiments could not be performed on the rhesus macaques. RNA was isolated from cell fractions using the Quick-RNA Miniprep kit (Zymo Research), and cDNA was synthesized using the RT2 First Strand Kit (Qiagen). Transcriptional activity in sorted cell populations was probed by qPCR using the RT2 qPCR Primer Assay (Qiagen) for ISG15, as well as the following primers for DDX58: For-5′-GGAAGACCCTGGACCCTACCT-3′; Rev-5′-AAAGCCACGGAACCAGCCTT-3′. CD14-sorted populations from a representative highly responding nondepleted animal (*C46456) and a representative minimally responding CD8-depleted animal (C18942) were selected for transcriptional profiling using the nCounter NHP Immunology Panel (NanoString).

### Flow cytometry and gating strategy

For absolute lymphocyte counts, whole blood was stained within 2 h of blood draw for the surface markers CD45 (PerCP; DO58-1283; BD Biosciences), CD3 (FITC; SP34; BD Biosciences), CD4 (APC; L200; BD Biosciences), and CD8 (V500; SK1; BD Biosciences). Flow cytometry was performed on a BD FACSVerse instrument, and absolute counts were calculated using FACS Suite software.

For immunophenotyping, PBMCs from the indicated timepoints were thawed, washed, and stained using Live/Dead Fixable Aqua Dead Cell Stain Kit (Invitrogen). PBMCs were then stained for the surface markers CD16 (AL488; 3G8; BioLegend), CD169 (PE; 7-239; BioLegend), CD28 (PECF594; CD28.2; BD Biosciences), CD95 (PCP-Cy5.5; DX2; BioLegend), CD3 (PE-Cy7; SP34-2; BD Biosciences), CD8 (PacBlue; SK1; BioLegend), CD14 (BV605; M5E2; BD Biosciences), HLA-DR (BV650; L243; BioLegend), NKG2A (APC; Z199; Beckman Coulter), and CD4 (APC-H7; L200; BD Biosciences). Cells were subsequently fixed in FluoroFix buffer (BioLegend), permeabilized using Perm/Wash buffer (BioLegend), and stained intracellularly for CD69 (BV711; FN50; BD Biosciences) and Ki67 (AL700; B56; BD Biosciences). Flow cytometry was performed on a BD LSRII instrument and data were analyzed using FlowJo (vX.10.4.2) and visual t-distributed stochastic neighbor embedding (viSNE) (Cytobank) softwares. For viSNE analysis, live singlet monocytes (CD14+ and/or CD16+) were gated prior to downsampling at a minimum of 500 cells per animal in FlowJo v. 10.5.3 for computational feasibility. Downsampled files for each animal were then concatenated by group (i.e., species, dpi, and treatment condition). When the number of animals differed per group, concatenated files were further downsampled to achieve an equal number of cells per group. viSNE was conducted using Cytobank with the following settings: Perplexity = 30, Iterations = 1,000, Theta = 0.5, Seed = random, Compensation = internal file. The following parameters were utilized in the run: Ki67, CD14, HLA-DR, CD69, CD95, CD14, and CD169.

For general immunophenotyping analysis, cytometry data were first gated for lymphocytes, singlets, and live cells. NK cells were considered as CD8+/CD16+. CD4 T cells (CD3+/CD4+) and CD8 T cells (CD3+/CD8+) were gated into naïve (CD28+/CD95−), central memory (CM) (CD28+/CD95+), and effector memory (EM) (CD28−/CD95+) subsets. CD3-cells were divided into B cells (DR+/CD14−/CD16−) and monocytes (classical, CD14++/CD16-; intermediate, CD14+/CD16+; nonclassical, CD14^low^/CD16+). Cell subsets were analyzed with respect to frequency, proliferation (Ki67+) and activation (CD69+ or CD169+).

### Intracellular cytokine staining

PBMCs from the indicated timepoints were thawed and rested overnight prior to stimulation with peptide pools comprising ZIKV capsid (C), membrane (M), envelope (E), and nonstructural protein 1 (NS1) (BEI Resources). On peptide stimulation, cells were also treated with brefeldin A (BioLegend), GolgiStop (BD Biosciences), anti-CD28 (NHP Reagent Reference Program, www.nhpreagents.org/), anti-CD49d (9F10; BioLegend), and anti-CD107a (AL700; H4A3; BD Biosciences). 24 h post-stimulation, cells were stained for the surface markers CD3 (PE-Cy7; SP34-2; BD Biosciences), CD8 (PacBlue; SK1; BioLegend), and CD4 (APC-H7; L200; BD Biosciences). Cells were also fixed and permeabilized as described above and stained intracellularly for perforin (FITC; Pf-344; Mabtech), granzyme B (PE; GB12; Invitrogen), CD69 (PE-CF594; FN50; BD Biosciences), IL-2 (PCP-Cy5.5; MQ1-17H12; BD Biosciences), and IFNγ (AL647; 4S.B3; BioLegend). Flow cytometry was performed on a BD LSRII instrument and data were analyzed using FlowJo software (vX.10.4.2).

### Plaque reduction neutralization tests

ZIKV plaque reduction neutralization tests (PRNTs) were conducted according to previously published protocols^18,19^. Briefly, ZIKV MEX-I-44 isolated in Tapachula, Mexico in 2016 was obtained from The University of Texas Medical Branch, Galveston, TX and cultured to passage 8 in Vero cells. Serum specimens were incubated for one hour at serial dilutions of 1:10, 1:20…1:320 with a previously frozen virus stock of known plaque forming unit (PFU). Samples were then inoculated in duplicate onto a mono-layer of Vero cells grown on 6-well plates and allowed to incubate for an additional hour. Infectious material was then removed and replaced with a 1:1 mixture of Vero media and Avicel before being incubated for 4 days. To read plaques, the Avicel layer was fixed with 10% neutral buffered formalin. Finally, the formalin-Avicel layer was removed and the monolayer was stained with crystal violet, washed with tap water and allowed to dry before plaques were counted manually.

Percent reduction in observed plaques and a PRNT90 cutoff were used for interpretation. A PRNT90 titer is the dilution of a sample at which a 90% reduction in possible plaques is observed. The maximum number of potential plaques was obtained for each run using a corresponding back-titration and a linear model was fit to the observed number of plaques for each dilution. A PRNT90 titer was derived for each sample using the linear model and the equation for a straight line in the statistical program R^20^. For samples that were positive but above the resolution of the PRNT assay the value of the greatest number of possible plaques for that run, as determined by the back titration, was assigned for each dilution for use with the linear model.

### Histology

Tissues samples collected at necropsy were fixed in Z-Fix (Anatech), embedded in paraffin and 5 μm thick sections were cut, adhered to charged glass slides, and either stained routinely with hematoxylin and eosin or Prussian blue.

### Statistical analysis

Statistical analysis was conducted using GraphPad Prism v8.2.1 (GraphPad Software). Mann–Whitney tests were used to compare viral loads and immunophenotypic patterns among CD8-depleted (n = 4) and nondepleted (n = 5) animals. Area-under-the-curve analysis was utilized in time-course measures.

## Results

### Viral loads and transcriptional responses

**Figure 1:**
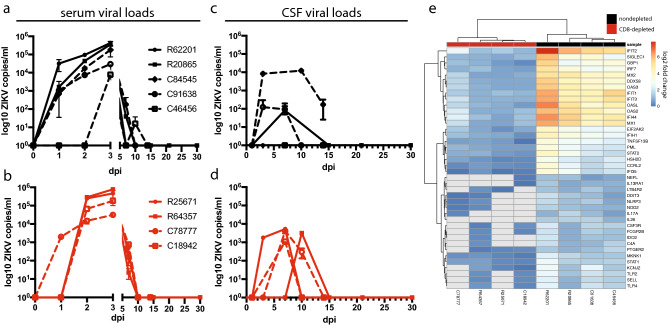
Viral loads and transcriptional responses. (**a**,**b**) Viral loads in the serum (**a**) and cerebrospinal fluid (CSF, **b**) in a cohort of rhesus (closed symbols, solid lines) and cynomolgus (open symbols, dashed lines) macaques (error bars, standard deviation, consistent throughout). (**c**,**d**) A second cohort of rhesus and cynomolgus macaques (shown in red) was depleted of CD8+ T cells, and viral loads in serum (**c**) and CSF (**d**) are shown. Differences in serum and CSF viral loads between CD8-depleted and nondepleted macaques were evaluated using Mann–Whitney tests of area-under-the-curve analysis, but the resulting *p* values were not significant. (**e**) Heatmap showing antiviral gene induction in whole blood at 3 dpi relative to preinfection. Legend shows nondepleted (black) and CD8-depleted (red) animals, and fold change gene expression values were calculated using the NanoString platform for immune system related genes. The 40 most differentially expressed genes between CD8-depleted and nondepleted animals are shown. The heatmap was generated using the pheatmap package (version 1.0.12, https://cran.r-project.org/web/packages/pheatmap/index.html) in R (version 3.6.1, https://cran.r-project.org/bin/windows/base/old/3.6.1/).

Following ZIKV challenge, rhesus and cynomolgus macaques showed rapid induction of viral loads in serum at 3–4.5 logs the day following challenge (Fig. 1a), consistent with previous reports of ZIKV infection in these species^9,13,14^. A single cynomolgus macaque (*C46456) did not have viral RNA in the serum until 3 days post infection (dpi) (Fig. 1a). However, this animal had been splenectomized in a prior study (denoted with an asterisk), and the spleen is a major site of replication of mosquito-borne flaviviruses related to ZIKV^21,22^ . In all animals, viral RNA peaked in the serum at 3 dpi and dropped to an undetectable level by 10 dpi and beyond, with exception to a small viral rebound in *C45656 (Fig. 1a). Two cynomolgus macaques and a single rhesus macaque showed virus in the cerebrospinal fluid (CSF) (Fig. 1b), although CSF was sampled less frequently than the blood. Viral RNA in the CSF was generally detected at lower levels compared to the serum and was similarly cleared past 7 dpi in two of three animals (Fig. 1b).

To evaluate the impact of CD8 T cells in controlling ZIKV infection, we depleted these cells prior to challenge in an additional two animals of each species by administration of an antibody specific to CD8α^16^ (targeting primarily CD8 T cells and NK cells). We also administered an irrelevant control antibody in a separate animal (C84545) to distinguish effects caused by antibody administration alone. CD8+ lymphocyte depletion commenced 14 days prior to ZIKV inoculation, and CD8 T cells and NK cells were undetectable in all depleted animals well before challenge (Fig. S1a–c). CD4/CD8 double-positive T cells were also depleted (Fig. S1d), while CD4 T cells were not (Fig. S1e). The CD8-depleted rhesus macaque R64357 recovered CD8 T cells and NK cells at later timepoints, between 15 and 21 dpi (Figs. S1b, c). Intriguingly, viral loads were not detected in the serum until 2 dpi in three of four CD8-depleted macaques, but similarly to nondepleted animals, viral RNA peaked at 3 dpi and was undetectable by 10 dpi (Fig. 1c). ZIKV was detected in the CSF of all four depleted animals, which was again 1–2 logs lower and peaked later at 7–10 dpi compared to the serum (Fig. 1d).

To survey antiviral signaling patterns in CD8-depleted and nondepleted animals, we carried out transcriptional analysis of immune responses in whole blood using the NanoString platform, which revealed striking differences in activation patterns among the cohorts (Fig. 1e). At 3 dpi, nondepleted rhesus and cynomolgus macaques showed strong induction of genes principally involved in interferon (IFN) signaling, while in CD8-depleted animals, we failed to detect induction of any genes included in the NanoString NHP Immunology panel (Fig. 1e). The most highly induced genes in nondepleted animals included innate immune sensors (*DDX58*, *IFIH1*, and several TLRs) as well as transcriptional regulators involved in IFN signaling (*IRF7*, *EIF2AK2*) and IFN stimulated genes (ISGs, *IFIT1/2/3*, *MX2*, *OAS2/3*).

### Monocyte transcriptional profiles and phenotyping

**Figure 2:**
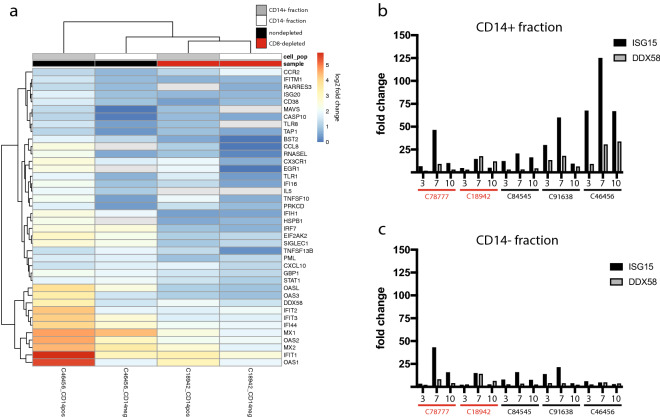
Monocyte transcriptional responses. (**a**) Heatmap showing patterns of antiviral gene expression in sorted CD14+ monocytes. Legend shows CD14+ (gray) and CD14− (white) fractions from the PBMCs of CD8-depleted and nondepleted animals, and fold change gene expression values were calculated using the NanoString platform for immune system related genes at 3 dpi relative to preinfection. The heatmap was generated using the pheatmap package (version 1.0.12, https://cran.r-project.org/web/packages/pheatmap/index.html) in R (version 3.6.1, https://cran.r-project.org/bin/windows/base/old/3.6.1/). (**b**,**c**) qPCR verification of antiviral gene expression in sorted CD14+ monocytes. Bar graphs shows the relative expression levels of an interferon stimulated gene (*ISG15*) and a pattern recognition related gene (*DDX58*) relative to b-actin in sorted CD14+ monocyte (**b**) and CD14− (**c**) fractions at the indicated timepoints (dpi, x-axis).

Given that monocytes are primary targets of ZIKV in the blood^23,24^ and contribute to antiviral signaling during ZIKV infection^25^, we carried out targeted transcriptional analysis in monocytes to assess the contribution of these cells to antiviral signaling patterns observed in the blood. NanoString analysis showed that monocytes sorted from peripheral blood mononuclear cells (PBMCs) of a nondepleted animal had a greater extent of antiviral gene expression compared to the CD14-fraction from the same animal and compared to both fractions from a CD8-depleted animal (Fig. 2a). From the non-depleted animals, sufficient cells were only available for C46456, the splenectomized animal, which may have led to data not representative of normal animals. However, we note that responses in this animal in whole blood (Fig. 1e), mirrored the other non-depleted animal, justifying the use of cells from this animal for this targeted experiment. Transcriptional patterns in the monocytes again centered on IFN signaling. To confirm these patterns and explore activation at additional timepoints, we sorted monocytes from all cynomolgus macaques at 3, 7, and 10 dpi and used qPCR to measure induction of representative antiviral response genes (*ISG15* and *DDX58*). Antiviral gene expression was highest in the monocyte fraction (CD14+) from nondepleted animals and peaked at 7 dpi (Fig. 2b), and CD8-depleted animals were again transcriptionally quiescent in both fractions (Fig. 2b,c). These data confirm patterns we observed using the NanoString platform and suggest that monocytes are contributing to the transcriptional patterns observed in whole blood.

**Figure 3:**
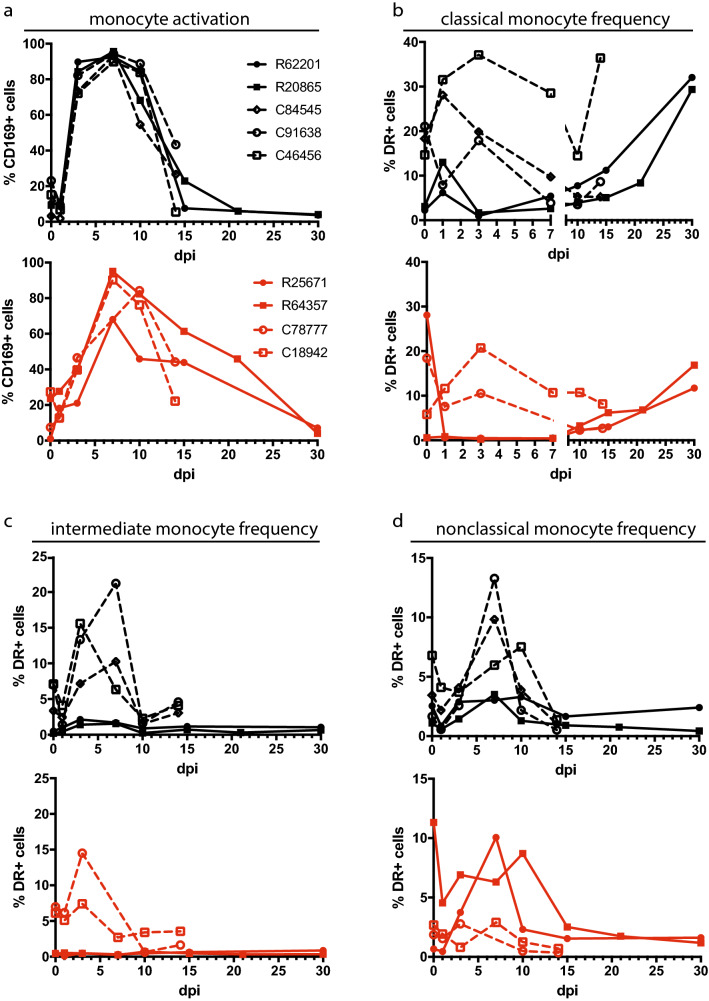
Monocyte phenotyping. (**a**) Flow cytometric analysis of monocyte activation by CD169 expression in nondepleted (black, top) and CD8-depleted (red, bottom) rhesus and cynomolgus macaques. (**b**–**d**) Flow cytometric analysis of monocyte subset frequencies. The percentages of total DR+ cells that were gated as classical (**b**), intermediate (**c**), and nonclassical (**d**) monocytes are plotted. Mann–Whitney tests of area-under-the-curve analysis between CD8-depleted and nondepleted macaques were not significant.

To evaluate the activation of monocytes phenotypically, we developed a multicolor flow cytometry panel that included CD169, a sialic acid binding lectin and inflammatory biomarker that has been used to assess monocyte activation during ZIKV infection in macaques^26–28^. CD169 is encoded by *SIGLEC-1*, which was one of the most highly induced genes in the whole blood of nondepleted animals (Fig. 1e). In line with the transcriptional data, nondepleted animals of both species showed early and robust activation of monocytes, with the percentage of CD169+ monocytes increasing sharply to 70–90% between 1 and 3 dpi (Fig. 3a). Meanwhile, monocytes in the CD8-depleted animals showed a minimal increase in CD169 expression over the same timeframe, and the overall kinetics of CD169 expression was less uniform relative to nondepleted animals (Fig. 3a). This disparity in monocyte activation was consistent in the classical (CD14++/CD16−), intermediate (CD14+/CD16+), and nonclassical (CD14^low^/CD16+) monocyte subsets (Fig. S2a–c). Monocyte frequencies also showed changes in frequency over the course of infection, but these patterns appeared to be attributed more to species differences than CD8 depletion. Although classical monocytes increased immediately following infection in 4 of 5 nondepleted macaques but only 1 of 4 CD8-depleted animals, the frequency of classical monocytes continued to rise until 3 dpi only in cynomolgus macaques, whereas rhesus macaques showed increases in classical monocyte frequency past 15 dpi, after the cynomolgus macaques had been sacrificed (Fig. 3b). The frequency of intermediate monocytes increased in cynomolgus macaques, peaking at 3–7 dpi (Fig. 3c), whereas nonclassical monocytes expanded over the same timeframe in all animals, but the greatest increases occurred in nondepleted cynomolgus macaques and CD8-depleted rhesus macaques (Fig. 3d).

### T cell and humoral responses

**Figure 4:**
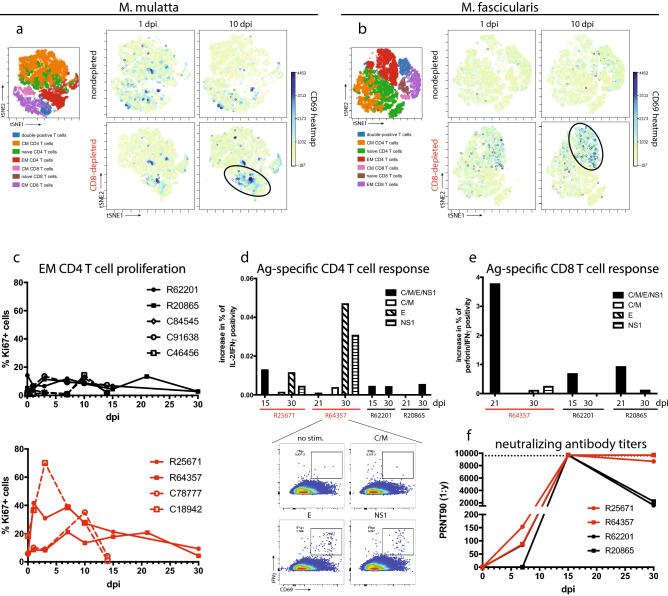
Adaptive immune responses. (**a**,**b**) viSNE analyses of T cell activation in rhesus (**a**) and cynomolgus (**b**) macaques, as measured by CD69 expression. Dot plots are concatenated for CD8-depleted and nondepleted animals within each species. The viSNE clustering profiles of CD4 and CD8 T cell subsets (*left*), including naïve, central memory (CM), and effector memory (EM) T cells correspond to cell populations in the CD69 heatmaps in nondepleted and CD8-depleted animals at 1 and 10 dpi (*right*). Black ovals indicate EM CD4 populations in CD8-depleted animals at 10 dpi, which showed higher levels of CD69 expression compared to nondepleted animals at the same timepoint. (**c**) Proliferation of EM CD4 T cells by Ki67 expression. Area-under-the-curve analysis revealed a significant difference in EM CD4 T cell proliferation among CD8-depleted (n = 4) and nondepleted (n = 5) animals by a Mann–Whitney test (*p* = 0.0159). viSNE plots were generated using viSNE software (Cytobank version 7.0, https://www.cytobank.org/platform.html). (**d**) CD4 T cell responses in rhesus macaques, assessed by intracellular cytokine staining (ICS) of PBMCs stimulated with viral peptides derived from the indicated ZIKV proteins (C = capsid; M = membrane; E = envelope; NS1 = nonstructural protein 1, consistent throughout). CD4 T cell responses were identified by co-positivity for IL-2 and IFNγ. (*Inset*): representative antigen-specific cytometry plots for R64357 (CD8-depleted) at 30 dpi. Dot plots were generated using FlowJo (version X.10.4.2, https://www.flowjo.com/). (**e**) CD8 T cell responses, determined by ICS for perforin and IFNγ co-positivity. (**f**) Serum neutralizing antibody titers in rhesus macaques, represented as PRNT90.

We also used flow cytometry to track the activation (CD69) and proliferation (Ki67) of CD4 and CD8 T cells, and we used the viSNE algorithm^29^ to survey phenotypic changes in the central memory (CM), effector memory (EM), and naïve T cell compartments. viSNE analysis revealed what appeared to be greater activation of EM CD4 T cells in CD8-depleted macaques compared to nondepleted animals (Fig. 4a,b and S3a), and this same cell population also showed significantly greater proliferation in CD8-depleted animals (Fig. 4c). CM and naïve CD4 T cells also showed increases in proliferation in both cohorts which was generally highest at 10 dpi (Fig. S3b,d). Activation of CM and naïve CD4 T cells occurred primarily in CD8-depleted animals and also peaked at 10 dpi (Fig. S3c,e).

To further interrogate antigen-specific T cell responses, we carried out intracellular cytokine staining (ICS) in T cells stimulated with overlapping peptide pools derived from the ZIKV proteins capsid (C), membrane (M), envelope (E), and nonstructural protein 1 (NS1). Corroborating the flow cytometry data, we detected CD4 T cell responses characterized by co-positivity for IL-2 and IFNγ which were marginally higher in the CD8-depleted rhesus macaques (Fig. 4d). Notably, these responses were specific for E and NS1. In nondepleted rhesus macaques and the one CD8-depleted animal that recovered CD8 cells (R64357), we identified IFNγ producing CD8 T cells that contained perforin at 15 and 21 dpi (Fig. 4e). We also observed phenotypic changes in CD8 T cell subsets in the nondepleted animals that largely mirrored trends that occurred in CD4 T cells. Proliferation of EM, CM, and naïve CD8 T cells occurred in all nondepleted rhesus and cynomolgus macaques and peaked at 10 dpi (Fig. S4a,c,e), and these cells also became activated primarily in rhesus macaques over the same timeframe (Fig. S4b,d,f).

To gauge humoral responses to ZIKV, we carried out a plaque reduction neutralization test (PRNT) using rhesus macaque sera, which showed induction of neutralizing antibodies in 3 of 4 animals by 7 dpi and all 4 animals by 15 dpi (Fig. 4f). By necropsy at 30 dpi, antibody titers had declined in the nondepleted animals but remained near the limit of quantification in the CD8-depleted animals.

### Tissue dissemination and neuropathology

**Figure 5:**
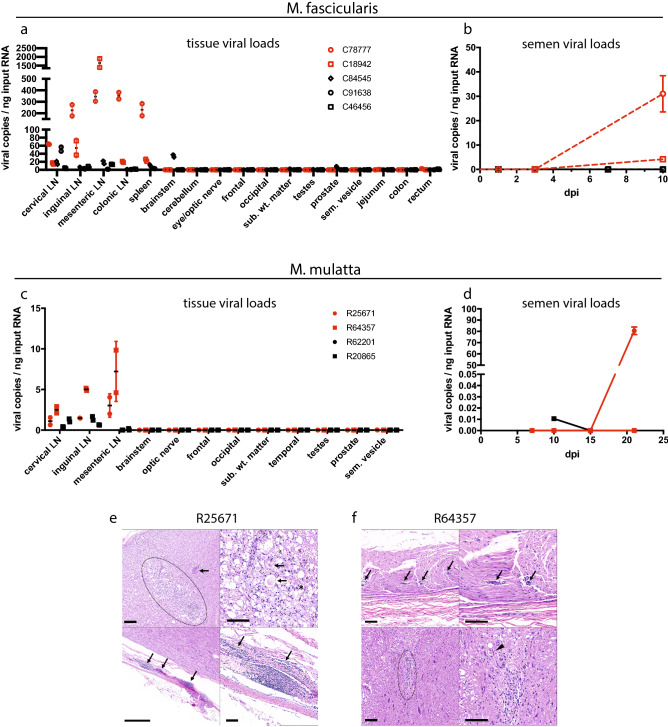
Tissue dissemination and neuropathology. (**a**) Viral dissemination in the lymphatic, neural, and reproductive tissues of cynomolgus macaques (center line, mean; error bars, standard deviation of two replicates per sample, LN = lymph node; sub. wt. matter = subcortical white matter, sem. vesicle = seminal vesicle, consistent throughout). Mann–Whitney tests comparing viral loads in each tissue among CD8-depleted and nondepleted animals returned non-significant *p* values. (**b**) Viral loads in semen during infection in cynomolgus macaques. (**c**) Viral dissemination in the lymphatic, neural, and reproductive tissues of rhesus macaques. Mann–Whitney tests comparing viral loads in each tissue among CD8-depleted and nondepleted animals returned non-significant *p* values. (**d**) Viral loads in semen during infection in rhesus macaques. (**e**) R25671 (CD8-depleted rhesus macaque) brainstem (top) and lumbar spinal cord (bottom). *Top*: there is an area of encephalomalacia (dotted region, left) adjacent to a vessel that exhibits medial thickening (arrow, left). The area of malacia is characterized by dilated myelin sheaths with swollen axons (arrow, right) or gitter cell infiltration (asterisks, right). H&E, Bar = 100 µm. *Bottom*: the meninges surrounding the lumbar spinal cord are multifocally infiltrated by aggregates of lymphocytes (arrows). H&E, Bar = 1 mm (left) and 100 µm (right). (**f**) R64357 (CD8-depleted rhesus macaque) sciatic nerve (top) and brainstem lesions (bottom). *Top*: small vessels within the sciatic nerve are surrounded by low numbers of lymphocytes (arrows). *Bottom*: a focal glial nodule is present within the gray matter of the brainstem (dotted region, left) with dilation of adjacent myelin sheaths and spheroid formation (arrowhead, right). H&E, Bar = 100 µm.

Given indications that ZIKV dissemination to neural and reproductive tissues contributes to neurologic disease and enhanced transmission dynamics^30,31^, and guided by previous reports of ZIKV tropism in macaques^11,13,32^, we searched for viral RNA in a variety of tissues and in semen to evaluate viral distribution in these sites. Relative to nondepleted animals, CD8-depleted cynomolgus macaques had higher levels of ZIKV RNA in the inguinal, mesenteric, and colonic lymph nodes, as well as in the spleen (Fig. 5a), although the small sample size precluded statistical significance. All cynomolgus monkeys except C91638 (nondepleted) harbored a low level of virus in the rectum (Fig. S5), although the only cynomolgus macaques with virus detected in the jejunum and in semen were the 2 CD8-depleted animals (Figs. 5b and S5). Intriguingly, the mock-depleted macaque C84545 showed the highest level of viral RNA in the prostate and was the only animal to present virus in the testes (Fig. S5), yet no ZIKV was detected in the semen of this animal (Fig. 5b). C84545 was also the only animal with virus detected in the brainstem or subcortical white matter (Figs. 5a and S5), and this animal also had a high and persistent level of ZIKV in the CSF (Fig. 1b).

The trend of higher viral burdens in the lymphatic tissues of CD8-depleted animals appeared to be consistent in rhesus macaques (Fig. 5c). However, tissue viral loads were generally much lower in rhesus macaques, possibly due to the longer duration of infection before necropsy and tissue collection. Although ZIKV RNA was not detected in the reproductive tissues of any rhesus macaques, virus was present in the semen of one nondepleted and one CD8-depleted animal, with a higher level in the CD8-depleted macaque (Fig. 5d). ZIKV was also not detected in the central nervous system (CNS) of any of the rhesus macaques, but both CD8-depleted animals (R25671 and R64357) manifested neural lesions at necropsy. Most strikingly, the brainstem of R25671 had an area of severe multifocal to coalescing encephalomalacia which showed evidence of Wallerian degeneration, characterized by vacuolation, swollen axons, and infiltration by lymphocytes and phagocytic gitter cells (Fig. 5e). Gitter cells were occasionally found within dilated myelin sheaths. Scant brown granular pigment (presumed hemosiderin) and a proliferative cerebral vessel adjacent to the malacia may indicate that the malacia was the result of a vascular event (thromboembolism, infarct, ischemia, etc.). Additionally, lymphocytic infiltrate was present in the meninges surrounding the lumbar spinal cord (Fig. 5e). No gross abnormalities were noted in R64357, although the sciatic nerve exhibited mild lymphocytic perivasculitis. The sciatic nerve is a known site of ZIKV replication in mice depleted of CD8 cells^6^. Further, the brainstem contained a localized area of gliosis, an indicator of CNS damage^33^^,^ and dilated myelin sheaths (Fig. 5f). A cause for these neural inflammatory lesions was not apparent by histology.

## Discussion

The rapid development of several ZIKV vaccine candidates shows promise for curbing future outbreaks, but a better understanding of the immune responses that are important for providing protection during natural infection will be pivotal in evaluating these vaccines. Macaque models have begun to show the importance of innate and adaptive responses in controlling infection, so our goal was to provide an additional descriptive account of immune responses that occur following infection with a Brazilian ZIKV strain. We also asked whether CD8 T cells have a role in controlling infection, owing to the importance of these cells in providing protection in mouse models^4–6^.

In contrast to similar CD8-depletion studies in macaques with simian immunodeficiency virus (SIV) challenge, which leads to uncontrolled viral replication^34,35^, the absence of CD8 cells did not overtly affect the control of ZIKV in serum. The marginal delay in serum viral loads in CD8-depleted macaques contrasted patterns observed by our own group and others^9,12^, but the small sample size of the present study limited meaningful statistical analysis. CD8-depleted macaques appeared to have a higher persistence of virus in the lymphatic tissues and semen, which might have resulted in the greater magnitude of neutralizing antibodies in these animals. However, the different durations of infection before necropsy in rhesus and cynomolgus macaques made it impossible to compare all CD8-depleted and nondepleted animals directly. Neural lesions were also evident at necropsy in CD8-depleted but not in nondepleted animals, but our inability to detect viral RNA in brain sections from these animals precludes the conclusion that these lesions were virus associated. The mock-depleted cynomolgus macaque C84545 was the only animal with ZIKV identified in the brain, and this animal also showed the highest and most persistent level of viral RNA in CSF, raising the possibility that antibody administration alone may have an indirect effect on viral persistence. Overall, these data do not link the absence of CD8 cells to worse disease outcomes; rather, our findings point to small trends in viral dynamics and dissemination following CD8 depletion that warrant follow up in a more comprehensive study.

In line with the rapid control of serum viremia in CD8-depleted and nondepleted animals, we detected phenotypic changes in several innate and adaptive immune cell subsets in both cohorts, suggesting balanced responses to infection. Monocytes in all animals showed high levels of activation soon after infection, but these patterns were most robust in nondepleted animals. Although a mechanism underlying the attenuated monocyte activation in CD8-depleted animals remains unclear, the collateral depletion of NK cells by CD8 depletion might have disrupted a dynamic crosstalk between NK cells and monocytes that is known to co-activate both cell types^36,37^. Additionally, CD8-depleted animals showed lower levels of transcriptional activity in monocytes, which might also be a consequence of such a crosstalk mechanism. Unlike patterns of monocyte activation, which appeared to be affected by CD8 depletion, we also observed transient increases in monocyte frequencies, but these changes seemed mainly dependent on the macaque species rather than the presence of CD8 cells. The immediate increase in classical monocytes may be analogous to the monocytosis that accompanies acute ZIKV replication in human patients^23^^,^ and the expansion of intermediate and nonclassical monocytes are consistent with the effects of ZIKV infection in human monocytes^23,38^, as monocytes are primary targets of ZIKV in the blood^39^.

Finally, we detected phenotypic changes in CD4 and CD8 T cells, implying some involvement of the adaptive immune system in controlling infection. CD8 T cell subsets showed increases in proliferation and activation in the nondepleted animals, and intriguingly, similar changes in CD4 T cells occurred primarily in CD8-depleted animals. These responses were antigen-specific and functional, and peptide stimulation experiments reinforced the finding of higher magnitude CD4 T cell responses in CD8-depleted animals. All rhesus macaques also appeared to mount efficient humoral responses, but neutralizing antibodies persisted longer in the CD8-depleted animals. Experiments in mice have shown the plasticity of protection among adaptive immune responses during ZIKV infection, where the depletion of CD4 T cells or CD8 T cells promotes other branches to control infection^40^, so perhaps a similar immune dynamic exists in macaques. There is also precedence in mice and in humans for CD8 T cell responses to ZIKV^5,6,41–43^, which seems to be the case in macaques as well, although these responses were not crucial for controlling viremia. We caution that we failed to detect antigen specific T cell responses in cynomolgus macaques, so further work is needed to confirm whether these responses are important in other NHP species.

In summary, our findings describe innate and adaptive immune responses that may be important in controlling acute ZIKV infection in NHPs. While in this model CD8 T cells appear to be dispensable for limiting viremia, these cells may have a role in restricting the dissemination of ZIKV in tissues where the virus could persist. If confirmed, these findings would implicate CD8 cells in the long-term control of ZIKV in nonhuman primates.

## Supplementary information

Supplementary Figures.

Supplementary Legends.

## Data Availability

The datasets generated during and/or analyzed during the current study are available from the corresponding author on reasonable request.
