# Supplementary material for: Immune outcomes of Zika virus infection in nonhuman primates
Source: Sci Rep. 2020 Aug 3;10:13069. doi: 10.1038/s41598-020-69978-w (PMC7400481; doi:10.1038/s41598-020-69978-w)

Immune outcomes of Zika virus infection in nonhuman primates

Blake Schouest, Marissa Fahlberg, Elizabeth A. Scheef, Matthew J. Ward, Kyra Headrick, Dawn M. Szeltner, Robert V. Blair, Margaret H. Gilbert, Lara A. Doyle-Meyers, Victoria W. Danner, Myrna C. Bonaldo, Dawn M. Wesson, Antonito T. Panganiban, Nicholas J. Maness

Supplementary Figure 1

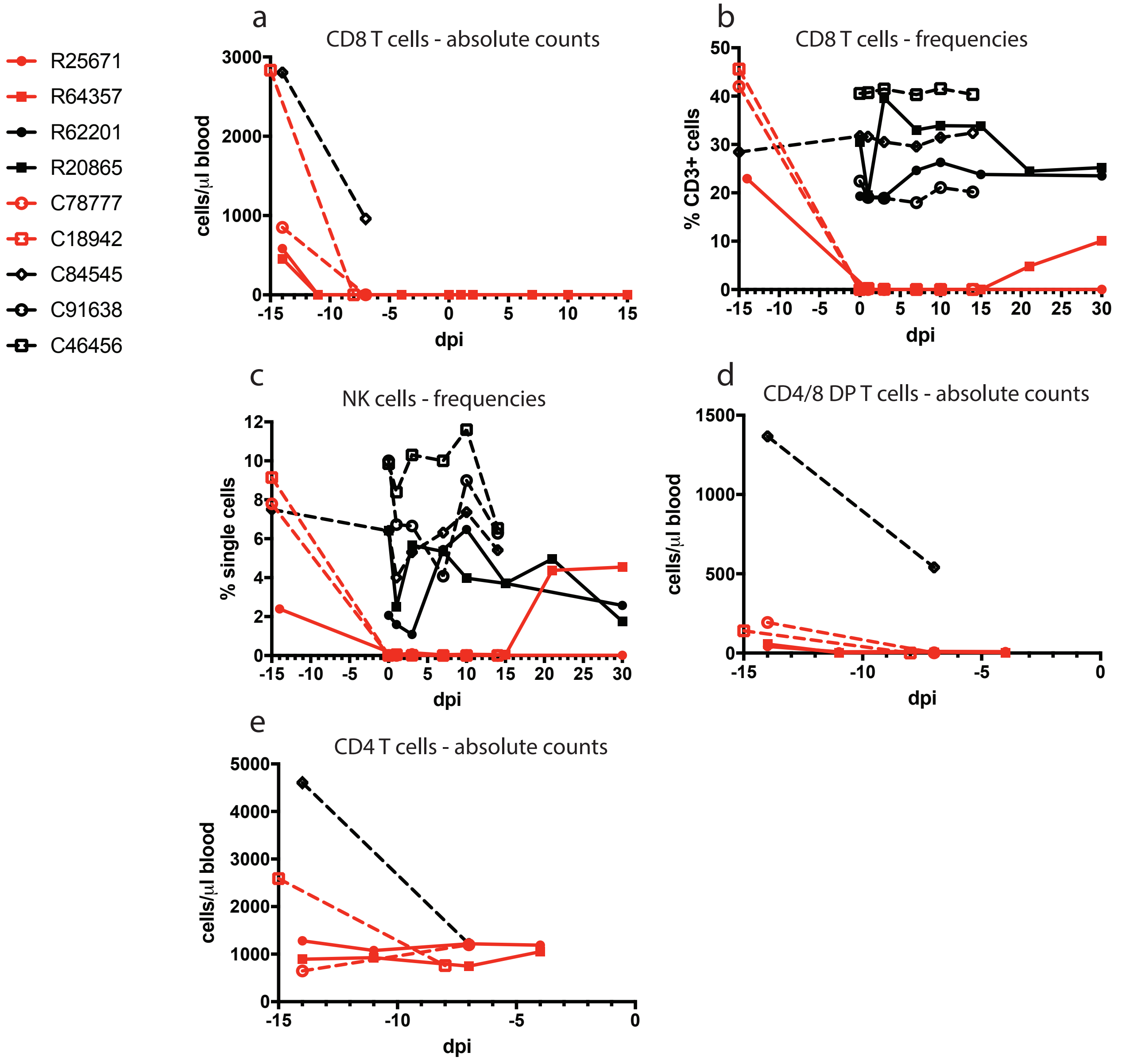

Supplementary Figure 2

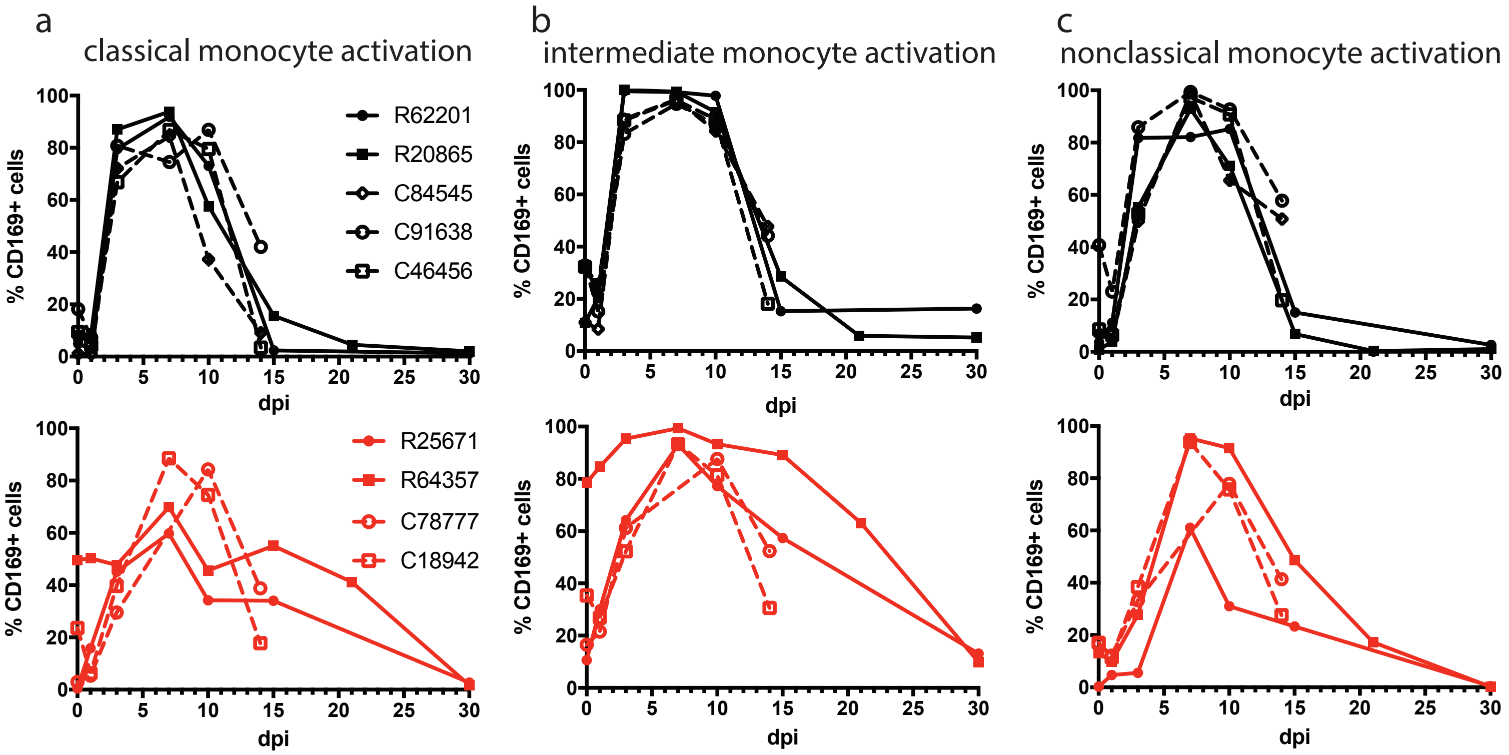

Supplementary Figure 3

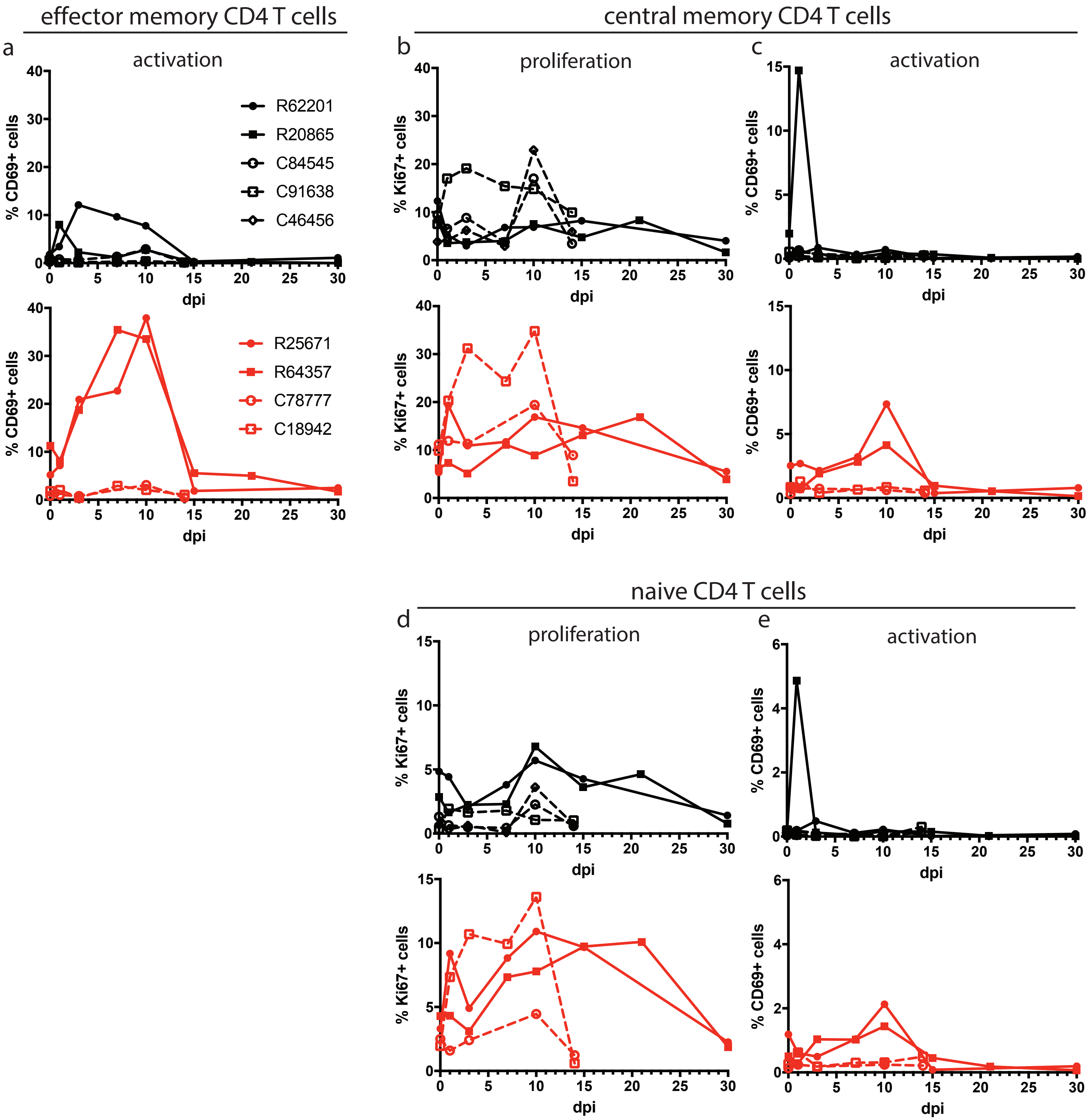

Supplementary Figure 4

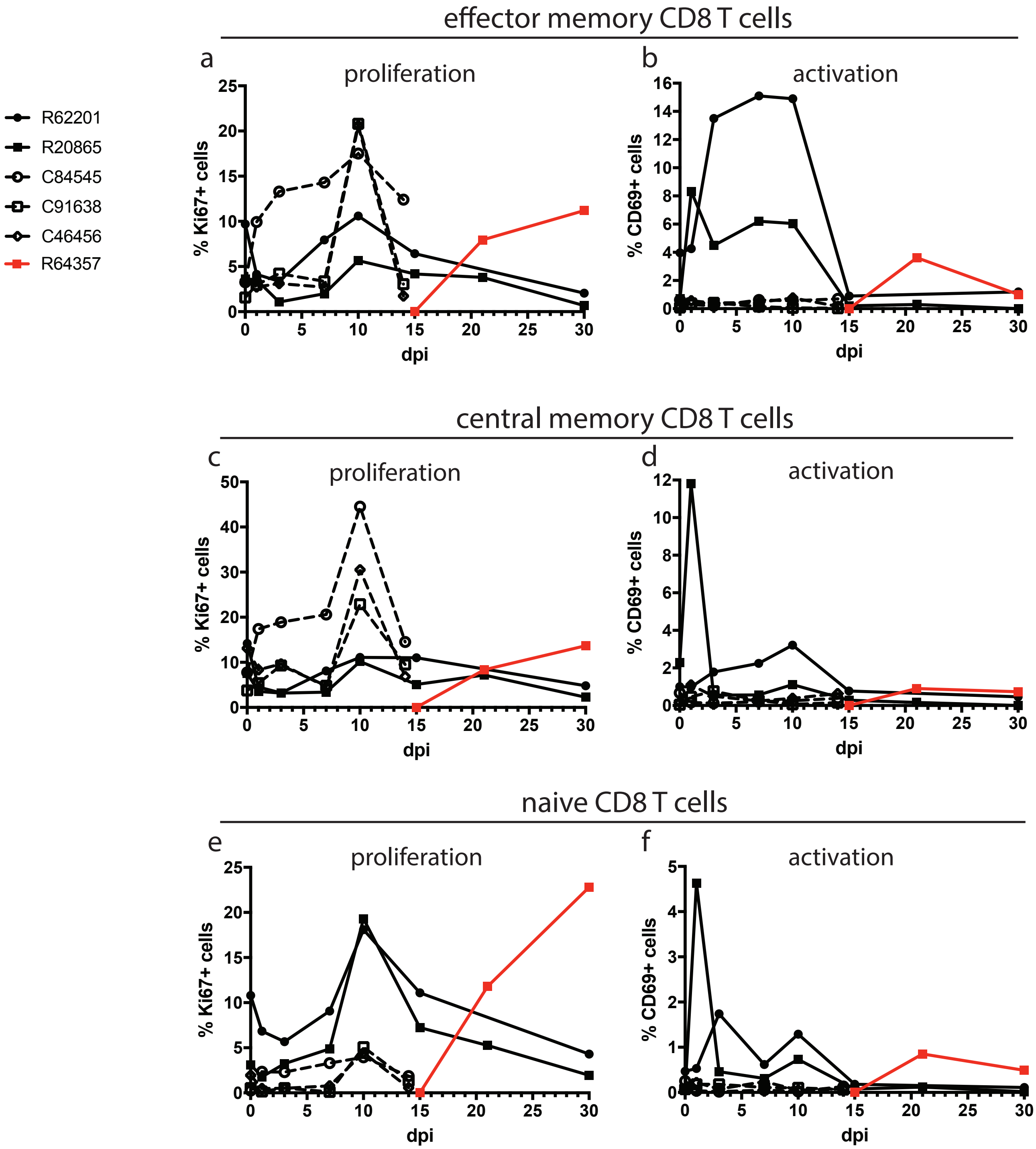

Supplementary Figure 5

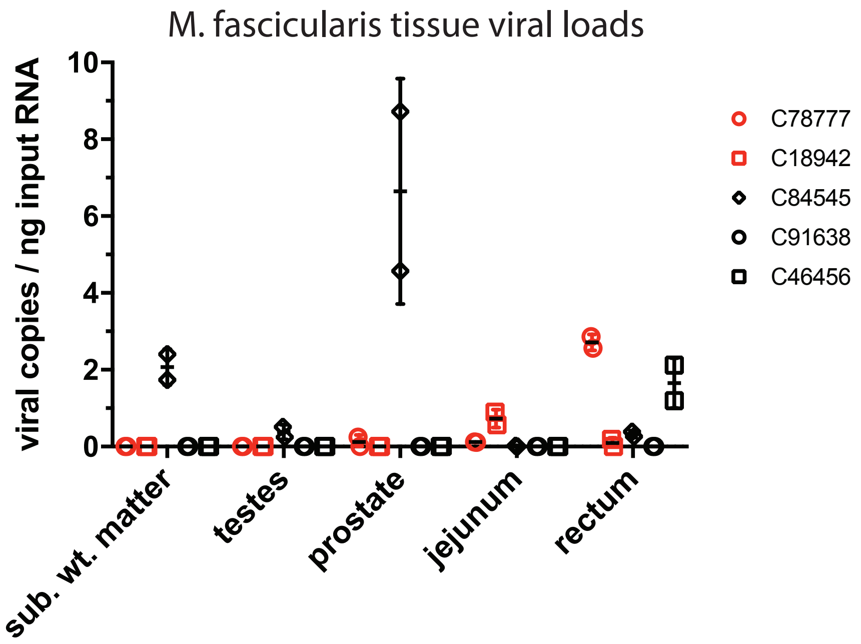

Supplement: Supplementary file 1 — Supplementary Figures. [file 41598_2020_69978_MOESM1_ESM.pdf]
