# Supplementary material for: Immune outcomes of Zika virus infection in nonhuman primates
Source: Sci Rep. 2020 Aug 3;10:13069. doi: 10.1038/s41598-020-69978-w (PMC7400481; doi:10.1038/s41598-020-69978-w)
Supplement: Supplementary file 2 — Supplementary Legends. [file 41598_2020_69978_MOESM2_ESM.docx]

**Supplementary Figure 1: CD8+ lymphocyte depletion**. Absolute counts (**A**) and frequencies (**B**) of CD8 T cells, frequencies of NK cells (**C**), and absolute counts of CD4/CD8 double-positive (DP) T cells (**D**) and CD4 T cells (**E**) in the blood. Absolute counts were obtained from complete blood count (CBC), and frequencies were calculated using flow cytometry.

**Supplementary Figure 2: Activation of monocyte subsets**. Flow cytometric analysis of monocyte activation by CD169 expression in the classical (**A**), intermediate (**B**), and nonclassical (**C**) subsets. Mann-Whitney tests of area-under-the-curve analysis between CD8-depleted and nondepleted macaques were not significant.

**Supplementary Figure 3: Phenotyping of CD4 T cell subsets**. Activation (CD69 expression) and proliferation (Ki67 expression) was assessed by flow cytometric analysis in effector memory (**A**), central memory (**B-C**), and naïve (**D-E**) CD4 T cells in CD8-depleted and nondepleted rhesus and cynomolgus macaques. Area-under-the-curve analysis revealed a significant difference in CM CD4 T cell proliferation among CD8-depleted (n=4) and nondepleted (n=5) animals by a Mann-Whitney test (p = 0.0317). All other comparisons of proliferation and activation of CD4 T cells subsets among CD8-depleted and nondepleted animals were not significant.

**Supplementary Figure 4: Phenotyping of CD8 T cell subsets**. Activation (CD69 expression) and proliferation (Ki67 expression) was assessed by flow cytometric analysis in effector memory (**A-B**), central memory (**C-D**), and naïve (**E-F**) CD8 T cells in nondepleted rhesus and cynomolgus macaques. Data from the CD8-depleted rhesus macaque R64357, which recovered CD8+ T cells, is also shown.

**Supplementary Figure 5: Low-level viral loads in M. fascicularis tissues**. Viral loads in the neural, reproductive, and GI tissues of cynomolgus macaques that harbored low levels of virus (center line, mean; error bars, standard deviation of two replicates per sample, sub. wt. matter = subcortical white matter).
